# (*E*)-*N*-(3,3-Di­phenyl­allyl­idene)naph­thal­en-1-amine

**DOI:** 10.1107/S1600536813014888

**Published:** 2013-06-08

**Authors:** Jae Kyun Lee, Kee Dal Nam, Joo Hwan Cha, Yong Seo Cho, Joon Kyun Lee

**Affiliations:** a Center for Neuro-Medicine, Brain Science Institute, Korea Institute of Science & Technology, Hwarangro 14-gil, Seongbuk-gu, Seoul 136-791, Republic of Korea; b Chemical Kinomics Research Center, Korea Institute of Science & Technology, Hwarangro 14-gil, Seongbuk-gu, Seoul 136-791, Republic of Korea; c Advanced Analysis Center, Korea Institute of Science & Technology, Hwarangro 14-gil, Seongbuk-gu, Seoul 136-791, Republic of Korea; d Korea Institute of Industrial Technology, 143 Hanggaulro, Sangnok-gu, Ansan-si, Gyeonggi-do 426-910, Republic of Korea

## Abstract

The title compound, C_25_H_19_N, adopts an *E* conformation about the C=N bond. The naphthalene ring system and the phenyl rings form dihedral angles 38.1 (1), 46.9 (8) and 48.5 (1)°, respectively, with the mean plane of the central enimino fragment. The crystal packing exhibits no directional close contacts.

## Related literature

For the crystal structures of related compounds studied recently by our group, see: Cha *et al.* (2012 ▶); Kang *et al.* (2012 ▶); Yu *et al.* (2013 ▶); Nam *et al.* (2013 ▶).
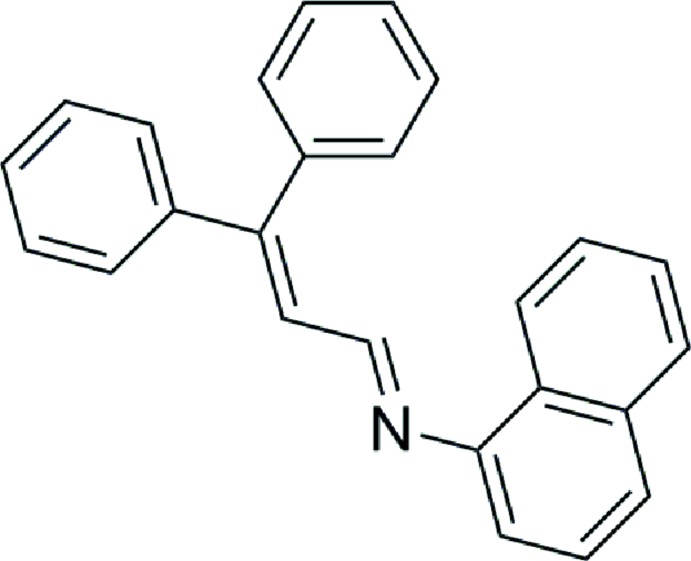


## Experimental

### 

#### Crystal data

C_25_H_19_N
*M* *_r_* = 333.41
Orthorhombic, 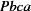

*a* = 11.2203 (7) Å
*b* = 13.5658 (7) Å
*c* = 24.1946 (13) Å
*V* = 3682.7 (4) Å^3^
*Z* = 8
Mo *K*α radiation
μ = 0.07 mm^−1^
*T* = 296 K
0.20 × 0.20 × 0.20 mm

#### Data collection

Rigaku R-AXIS RAPID diffractometer
Absorption correction: multi-scan (*ABSCOR*; Rigaku, 1995 ▶) *T*_min_ = 0.986, *T*_max_ = 0.986
33578 measured reflections
4190 independent reflections
2080 reflections with *I* > 2σ(*I*)
*R*_int_ = 0.070

#### Refinement

*R*[*F* ^2^ > 2σ(*F* ^2^)] = 0.061
*wR*(*F* ^2^) = 0.197
*S* = 0.95
4190 reflections
235 parameters
H-atom parameters constrained
Δρ_max_ = 0.16 e Å^−3^
Δρ_min_ = −0.25 e Å^−3^

### 

Data collection: *RAPID-AUTO* (Rigaku, 2006 ▶); cell refinement: *RAPID-AUTO*; data reduction: *RAPID-AUTO*; program(s) used to solve structure: *SHELXS97* (Sheldrick, 2008 ▶); program(s) used to refine structure: *SHELXL97* (Sheldrick, 2008 ▶); molecular graphics: *CrystalStructure* (Rigaku, 2010 ▶); software used to prepare material for publication: *CrystalStructure*.

## Supplementary Material

Crystal structure: contains datablock(s) I, global. DOI: 10.1107/S1600536813014888/cv5411sup1.cif

Structure factors: contains datablock(s) I. DOI: 10.1107/S1600536813014888/cv5411Isup2.hkl

Supplementary material file. DOI: 10.1107/S1600536813014888/cv5411Isup3.cml

Additional supplementary materials:  crystallographic information; 3D view; checkCIF report

## Figures and Tables

**Fig. 1.**
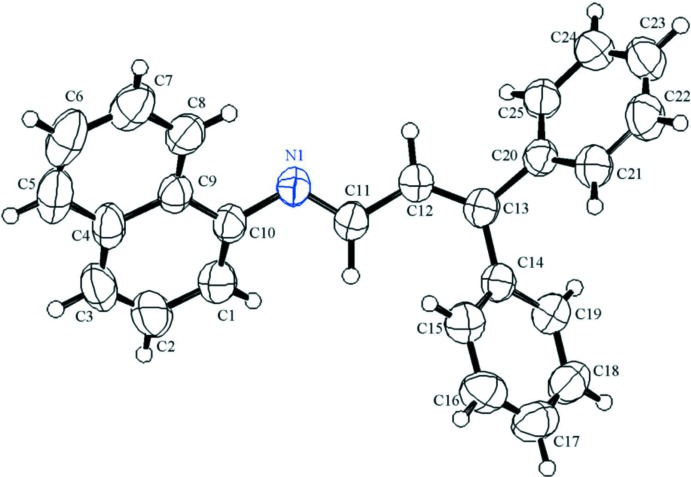
The molecular structure of the title compound showing the atomic numbering and 50% probability displacement ellipsoid.

## supplementary crystallographic information

### Comment

As a part of our ongoing study of 2-phenylcinnamaldehyde derivatives contaning aniline (Cha *et al.*, 2012; Kang *et al.*, 2012; Yu *et al.*, 2013) and naphthylamine (Nam *et al.*, 2013), we present here the title compound.

The title compound (Fig. 1) adopts an (*E*) conformation about the C═N bond. The naphthalene bicycle C1–C10 and phenyl rings C14–C19 and C20–C25 form the dihedral angles 38.1 (1), 46.9 (8) and 48.5 (1)°, respectively, with the mean plane of the central N1–C11–C12 enimino fragment. The crystal packing exhibits no classical intermolecular contacts.

### Experimental

In a solution of 1-naphthylamine (2.0 mmol) in anhydrous ethanol (50 mL) was treated with equimolar quantities of substituted 2-phenylcinnamaldehydes. The mixture was refluxed for 2 days, and the progress of reaction was monitored by TLC. After completion of reaction, the solvent was removed under reduced pressure. The residue was purified by flash column chromatography to afford the title compound as a yellow solid in yield 92%. Single crystals suitable for X-ray diffraction were prepared by slow evaporation of a solution of the title compound in ethanol at room temperature.

### Refinement

All hydrogen atoms were positioned geometrically (C—H = 0.93 Å), and refined using a riding model, with *U*_iso_(H) = 1.2 *U*_eq_(C).

### Crystal data

**Table:**

C_25_H_19_N	*Z* = 8
*M**_r_* = 333.41	*F*(000) = 1408
Orthorhombic, *P**b**c**a*	*D*_x_ = 1.203 Mg m^−^^3^
Hall symbol: -P 2ac 2ab	Mo *K*α radiation, λ = 0.71075 Å
*a* = 11.2203 (7) Å	µ = 0.07 mm^−^^1^
*b* = 13.5658 (7) Å	*T* = 296 K
*c* = 24.1946 (13) Å	Block, yellow
*V* = 3682.7 (4) Å^3^	0.20 × 0.20 × 0.20 mm

### Data collection

**Table:**

Rigaku R-AXIS RAPID diffractometer	4190 independent reflections
Radiation source: fine-focus sealed tube	2080 reflections with *I* > 2σ(*I*)
Graphite monochromator	*R*_int_ = 0.070
Detector resolution: 10.000 pixels mm^-1^	θ_max_ = 27.4°, θ_min_ = 3.0°
ω scans	*h* = −14→14
Absorption correction: multi-scan (*ABSCOR*; Rigaku, 1995)	*k* = −17→17
*T*_min_ = 0.986, *T*_max_ = 0.986	*l* = −31→30
33578 measured reflections	

### Refinement

**Table:**

Refinement on *F*^2^	Primary atom site location: structure-invariant direct methods
Least-squares matrix: full	Secondary atom site location: difference Fourier map
*R*[*F*^2^ > 2σ(*F*^2^)] = 0.061	Hydrogen site location: inferred from neighbouring sites
*wR*(*F*^2^) = 0.197	H-atom parameters constrained
*S* = 0.95	*w* = 1/[σ^2^(*F*_o_^2^) + (0.1186*P*)^2^] where *P* = (*F*_o_^2^ + 2*F*_c_^2^)/3
4190 reflections	(Δ/σ)_max_ = 0.001
235 parameters	Δρ_max_ = 0.16 e Å^−^^3^
0 restraints	Δρ_min_ = −0.25 e Å^−^^3^

### Special details

**Table:**

Geometry. All esds (except the esd in the dihedral angle between two l.s. planes) are estimated using the full covariance matrix. The cell esds are taken into account individually in the estimation of esds in distances, angles and torsion angles; correlations between esds in cell parameters are only used when they are defined by crystal symmetry. An approximate (isotropic) treatment of cell esds is used for estimating esds involving l.s. planes.
Refinement. Refinement of F^2^ against ALL reflections. The weighted R-factor wR and goodness of fit S are based on F^2^, conventional R-factors R are based on F, with F set to zero for negative F^2^. The threshold expression of F^2^ > 2sigma(F^2^) is used only for calculating R-factors(gt) etc. and is not relevant to the choice of reflections for refinement. R-factors based on F^2^ are statistically about twice as large as those based on F, and R- factors based on ALL data will be even larger.

### Fractional atomic coordinates and isotropic or equivalent isotropic displacement parameters (Å^2^)

**Table:**

	*x*	*y*	*z*	*U*_iso_*/*U*_eq_	
N1	0.03605 (16)	0.37399 (12)	0.86987 (6)	0.0588 (5)	
C1	−0.1297 (2)	0.27203 (18)	0.83686 (9)	0.0688 (6)	
H1	−0.1143	0.2251	0.8640	0.083*	
C2	−0.2224 (3)	0.2562 (2)	0.79886 (10)	0.0830 (8)	
H2	−0.2682	0.1992	0.8013	0.100*	
C3	−0.2458 (2)	0.3231 (2)	0.75873 (9)	0.0827 (8)	
H3	−0.3079	0.3118	0.7341	0.099*	
C4	−0.1772 (2)	0.4100 (2)	0.75384 (8)	0.0682 (7)	
C5	−0.1976 (3)	0.4814 (3)	0.71211 (10)	0.0888 (9)	
H5	−0.2569	0.4705	0.6860	0.107*	
C6	−0.1321 (3)	0.5650 (3)	0.70969 (10)	0.0947 (10)	
H6	−0.1471	0.6108	0.6819	0.114*	
C7	−0.0421 (3)	0.5836 (2)	0.74842 (11)	0.0850 (8)	
H7	0.0018	0.6416	0.7465	0.102*	
C8	−0.0190 (2)	0.51646 (18)	0.78898 (9)	0.0690 (7)	
H8	0.0406	0.5295	0.8147	0.083*	
C9	−0.0839 (2)	0.42754 (17)	0.79262 (8)	0.0582 (6)	
C10	−0.06209 (19)	0.35522 (16)	0.83444 (8)	0.0564 (5)	
C11	0.0290 (2)	0.35090 (15)	0.92139 (8)	0.0558 (5)	
H11	−0.0394	0.3213	0.9354	0.067*	
C12	0.1286 (2)	0.37159 (14)	0.95689 (8)	0.0562 (5)	
H12	0.1979	0.3923	0.9393	0.067*	
C13	0.13445 (18)	0.36485 (14)	1.01268 (8)	0.0522 (5)	
C14	0.03384 (19)	0.33393 (15)	1.04830 (8)	0.0534 (5)	
C15	−0.0793 (2)	0.37540 (16)	1.04349 (9)	0.0630 (6)	
H15	−0.0937	0.4227	1.0165	0.076*	
C16	−0.1706 (2)	0.3467 (2)	1.07863 (10)	0.0761 (7)	
H16	−0.2457	0.3750	1.0751	0.091*	
C17	−0.1506 (3)	0.2765 (2)	1.11871 (10)	0.0793 (8)	
H17	−0.2122	0.2571	1.1420	0.095*	
C18	−0.0399 (3)	0.23538 (18)	1.12413 (9)	0.0747 (7)	
H18	−0.0264	0.1881	1.1513	0.090*	
C19	0.0517 (2)	0.26348 (16)	1.08965 (8)	0.0642 (6)	
H19	0.1266	0.2351	1.0939	0.077*	
C20	0.24624 (19)	0.39592 (14)	1.04092 (8)	0.0522 (5)	
C21	0.2411 (2)	0.45116 (16)	1.08939 (8)	0.0655 (6)	
H21	0.1676	0.4635	1.1058	0.079*	
C22	0.3434 (3)	0.48772 (19)	1.11332 (10)	0.0760 (7)	
H22	0.3385	0.5257	1.1452	0.091*	
C23	0.4527 (3)	0.46821 (19)	1.09016 (10)	0.0781 (7)	
H23	0.5216	0.4933	1.1062	0.094*	
C24	0.4600 (2)	0.41132 (18)	1.04309 (10)	0.0702 (6)	
H24	0.5340	0.3969	1.0279	0.084*	
C25	0.3577 (2)	0.37575 (16)	1.01848 (8)	0.0604 (6)	
H25	0.3634	0.3379	0.9865	0.072*	

### Atomic displacement parameters (Å^2^)

**Table:**

	*U*^11^	*U*^22^	*U*^33^	*U*^12^	*U*^13^	*U*^23^
N1	0.0562 (11)	0.0734 (12)	0.0467 (9)	0.0012 (9)	−0.0046 (8)	0.0029 (8)
C1	0.0738 (16)	0.0759 (15)	0.0568 (12)	−0.0053 (13)	−0.0020 (11)	−0.0047 (11)
C2	0.0819 (19)	0.0996 (19)	0.0675 (15)	−0.0148 (15)	−0.0064 (14)	−0.0201 (14)
C3	0.0614 (16)	0.127 (2)	0.0593 (13)	0.0046 (16)	−0.0104 (12)	−0.0266 (15)
C4	0.0574 (15)	0.1024 (18)	0.0447 (11)	0.0215 (13)	−0.0033 (10)	−0.0105 (12)
C5	0.080 (2)	0.133 (3)	0.0530 (13)	0.042 (2)	−0.0055 (13)	−0.0045 (15)
C6	0.108 (3)	0.119 (2)	0.0568 (15)	0.052 (2)	0.0038 (15)	0.0169 (16)
C7	0.087 (2)	0.0966 (18)	0.0716 (15)	0.0220 (15)	0.0105 (14)	0.0225 (14)
C8	0.0638 (15)	0.0851 (17)	0.0580 (13)	0.0107 (13)	0.0052 (11)	0.0094 (12)
C9	0.0501 (13)	0.0809 (15)	0.0436 (10)	0.0106 (11)	0.0032 (9)	−0.0022 (10)
C10	0.0531 (13)	0.0728 (14)	0.0432 (10)	0.0037 (11)	−0.0006 (9)	−0.0030 (9)
C11	0.0597 (13)	0.0604 (12)	0.0473 (10)	−0.0005 (10)	−0.0016 (9)	0.0012 (9)
C12	0.0552 (13)	0.0609 (13)	0.0524 (11)	−0.0005 (10)	−0.0043 (10)	0.0020 (9)
C13	0.0575 (13)	0.0496 (11)	0.0494 (10)	0.0035 (9)	−0.0017 (9)	−0.0002 (9)
C14	0.0587 (13)	0.0538 (11)	0.0477 (10)	0.0004 (10)	−0.0015 (9)	−0.0044 (9)
C15	0.0639 (15)	0.0627 (13)	0.0623 (12)	0.0056 (11)	0.0012 (11)	−0.0043 (10)
C16	0.0628 (16)	0.0831 (16)	0.0824 (16)	0.0050 (13)	0.0132 (13)	−0.0138 (14)
C17	0.083 (2)	0.0854 (18)	0.0691 (14)	−0.0109 (15)	0.0213 (14)	−0.0095 (13)
C18	0.0897 (19)	0.0763 (16)	0.0580 (13)	−0.0081 (14)	0.0090 (13)	0.0040 (11)
C19	0.0681 (15)	0.0682 (13)	0.0563 (12)	−0.0009 (11)	−0.0006 (11)	0.0054 (10)
C20	0.0568 (13)	0.0519 (11)	0.0480 (10)	−0.0005 (10)	−0.0030 (9)	0.0045 (8)
C21	0.0706 (15)	0.0692 (14)	0.0568 (12)	−0.0008 (12)	−0.0029 (11)	−0.0056 (10)
C22	0.089 (2)	0.0747 (16)	0.0643 (14)	−0.0092 (14)	−0.0104 (14)	−0.0122 (12)
C23	0.0777 (19)	0.0796 (16)	0.0770 (16)	−0.0184 (14)	−0.0201 (14)	0.0043 (13)
C24	0.0618 (15)	0.0793 (16)	0.0696 (14)	−0.0057 (12)	−0.0055 (12)	0.0098 (12)
C25	0.0607 (14)	0.0658 (13)	0.0547 (12)	0.0016 (11)	−0.0020 (10)	0.0003 (10)

### Geometric parameters (Å, º)

**Table:**

N1—C11	1.288 (2)	C13—C14	1.481 (3)
N1—C10	1.419 (3)	C13—C20	1.489 (3)
C1—C10	1.361 (3)	C14—C15	1.394 (3)
C1—C2	1.405 (3)	C14—C19	1.398 (3)
C1—H1	0.9300	C15—C16	1.387 (3)
C2—C3	1.355 (4)	C15—H15	0.9300
C2—H2	0.9300	C16—C17	1.377 (4)
C3—C4	1.412 (4)	C16—H16	0.9300
C3—H3	0.9300	C17—C18	1.368 (4)
C4—C5	1.417 (4)	C17—H17	0.9300
C4—C9	1.425 (3)	C18—C19	1.378 (3)
C5—C6	1.353 (4)	C18—H18	0.9300
C5—H5	0.9300	C19—H19	0.9300
C6—C7	1.400 (4)	C20—C25	1.391 (3)
C6—H6	0.9300	C20—C21	1.393 (3)
C7—C8	1.364 (3)	C21—C22	1.379 (3)
C7—H7	0.9300	C21—H21	0.9300
C8—C9	1.412 (3)	C22—C23	1.373 (3)
C8—H8	0.9300	C22—H22	0.9300
C9—C10	1.430 (3)	C23—C24	1.378 (3)
C11—C12	1.437 (3)	C23—H23	0.9300
C11—H11	0.9300	C24—C25	1.380 (3)
C12—C13	1.355 (3)	C24—H24	0.9300
C12—H12	0.9300	C25—H25	0.9300
			
C11—N1—C10	119.57 (18)	C12—C13—C20	118.61 (19)
C10—C1—C2	120.7 (2)	C14—C13—C20	117.07 (16)
C10—C1—H1	119.6	C15—C14—C19	117.8 (2)
C2—C1—H1	119.6	C15—C14—C13	122.11 (19)
C3—C2—C1	120.7 (3)	C19—C14—C13	120.05 (19)
C3—C2—H2	119.7	C16—C15—C14	120.5 (2)
C1—C2—H2	119.7	C16—C15—H15	119.7
C2—C3—C4	120.9 (2)	C14—C15—H15	119.7
C2—C3—H3	119.6	C17—C16—C15	120.4 (2)
C4—C3—H3	119.6	C17—C16—H16	119.8
C3—C4—C5	122.8 (2)	C15—C16—H16	119.8
C3—C4—C9	119.0 (2)	C18—C17—C16	119.8 (2)
C5—C4—C9	118.2 (3)	C18—C17—H17	120.1
C6—C5—C4	121.1 (3)	C16—C17—H17	120.1
C6—C5—H5	119.5	C17—C18—C19	120.4 (2)
C4—C5—H5	119.5	C17—C18—H18	119.8
C5—C6—C7	120.9 (3)	C19—C18—H18	119.8
C5—C6—H6	119.6	C18—C19—C14	121.0 (2)
C7—C6—H6	119.6	C18—C19—H19	119.5
C8—C7—C6	119.9 (3)	C14—C19—H19	119.5
C8—C7—H7	120.0	C25—C20—C21	118.2 (2)
C6—C7—H7	120.0	C25—C20—C13	121.51 (17)
C7—C8—C9	121.1 (2)	C21—C20—C13	120.2 (2)
C7—C8—H8	119.4	C22—C21—C20	120.8 (2)
C9—C8—H8	119.4	C22—C21—H21	119.6
C8—C9—C4	118.7 (2)	C20—C21—H21	119.6
C8—C9—C10	122.8 (2)	C23—C22—C21	120.2 (2)
C4—C9—C10	118.5 (2)	C23—C22—H22	119.9
C1—C10—N1	123.73 (19)	C21—C22—H22	119.9
C1—C10—C9	120.2 (2)	C22—C23—C24	119.9 (2)
N1—C10—C9	115.94 (19)	C22—C23—H23	120.1
N1—C11—C12	118.9 (2)	C24—C23—H23	120.1
N1—C11—H11	120.6	C23—C24—C25	120.2 (2)
C12—C11—H11	120.6	C23—C24—H24	119.9
C13—C12—C11	128.3 (2)	C25—C24—H24	119.9
C13—C12—H12	115.8	C24—C25—C20	120.7 (2)
C11—C12—H12	115.8	C24—C25—H25	119.7
C12—C13—C14	124.21 (19)	C20—C25—H25	119.7
			
C10—C1—C2—C3	0.5 (4)	C11—C12—C13—C20	−176.53 (18)
C1—C2—C3—C4	0.5 (4)	C12—C13—C14—C15	−49.7 (3)
C2—C3—C4—C5	179.2 (2)	C20—C13—C14—C15	126.6 (2)
C2—C3—C4—C9	−1.3 (3)	C12—C13—C14—C19	132.6 (2)
C3—C4—C5—C6	178.1 (2)	C20—C13—C14—C19	−51.1 (3)
C9—C4—C5—C6	−1.4 (4)	C19—C14—C15—C16	−0.4 (3)
C4—C5—C6—C7	0.0 (4)	C13—C14—C15—C16	−178.12 (19)
C5—C6—C7—C8	0.5 (4)	C14—C15—C16—C17	−0.2 (3)
C6—C7—C8—C9	0.4 (4)	C15—C16—C17—C18	0.4 (4)
C7—C8—C9—C4	−1.7 (3)	C16—C17—C18—C19	−0.2 (4)
C7—C8—C9—C10	179.8 (2)	C17—C18—C19—C14	−0.3 (4)
C3—C4—C9—C8	−177.3 (2)	C15—C14—C19—C18	0.6 (3)
C5—C4—C9—C8	2.2 (3)	C13—C14—C19—C18	178.42 (19)
C3—C4—C9—C10	1.2 (3)	C12—C13—C20—C25	−38.9 (3)
C5—C4—C9—C10	−179.31 (19)	C14—C13—C20—C25	144.7 (2)
C2—C1—C10—N1	−177.1 (2)	C12—C13—C20—C21	137.5 (2)
C2—C1—C10—C9	−0.6 (3)	C14—C13—C20—C21	−38.9 (3)
C11—N1—C10—C1	−40.3 (3)	C25—C20—C21—C22	2.3 (3)
C11—N1—C10—C9	143.1 (2)	C13—C20—C21—C22	−174.3 (2)
C8—C9—C10—C1	178.2 (2)	C20—C21—C22—C23	−1.4 (4)
C4—C9—C10—C1	−0.2 (3)	C21—C22—C23—C24	−0.5 (4)
C8—C9—C10—N1	−5.0 (3)	C22—C23—C24—C25	1.4 (4)
C4—C9—C10—N1	176.55 (18)	C23—C24—C25—C20	−0.5 (3)
C10—N1—C11—C12	−179.19 (18)	C21—C20—C25—C24	−1.3 (3)
N1—C11—C12—C13	171.0 (2)	C13—C20—C25—C24	175.22 (19)
C11—C12—C13—C14	−0.3 (3)		
